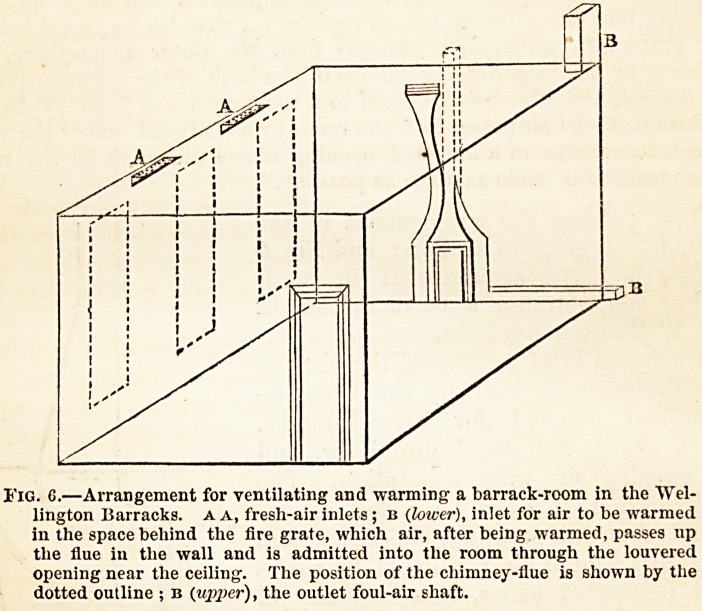# The Health of the Army, Prospective and Retrospective

**Published:** 1862-04

**Authors:** 


### Art. II. — THE HEALTH OF THE ARMY, PROSPECTIVE AND RETROSPECTIVE.

The War-Office and the Horse-Guards may justly take credit to themselves for the prompt and effective manner in which they have reinforced the garrison of Canada. The need was urgent, the season unpropitious; and supposing the Atlantic, when most storm-ridden, safely traversed, there were still to be contended with the grave obstacles which the early Canadian winter would interpose between the ports of debarkation and the posts of danger. Yet, notwithstanding these serious impediments, 15,000 men were thrown into the colony in a singularly short space of time, with all that was needful for their efficiency in artillery and stores, all that was necessary for their proper care in health or in sickness. We confess that, at the first, we felt far from assured that the sanitary efficiency of the soldier would meet with such care from the powers that be as would be at all comparable to its importance, or to the care which would be devoted to his military efficiency. The New Code of Medical Regulations, although founded on the terrible experience of the Crimean campaign, did not set our mind altogether at ease. The marvellous state of health of the troops engaged in the late Chinese war—a state largely due to the sanitary measures adopted in accordance with the requirements of the New Code, and so remarkable, that during the war, the *mortality*, exclusive of those killed in action or who died of their wounds, did not reach 4 (3·8) per cent. among the Europeans, and was below 3 (2·7) per cent. among the natives of India; while the mean sick among the former did not average 5 (4·6) per cent., and among the latter was but a fraction above 3 (3·1) per cent.*—even this success failed to give us confidence. The success, indeed, proved one thing to demonstration—the admirable capability of the Medical Department to do its work most thoroughly, now that it was no longer trammelled by that meaningless, aimless routine which had played so baneful a part in the disastrous winter of 1854-55. Certainly there was the consolatory thought that the men under whose direction the medical arrangements for the Chinese war had been carried to so successful a conclusion, had still the control of the medical affairs of the army: Dr. Gibson was still Director-General, the heads of his Staff were still the same. We had no doubt, in fact, that the Medical Department would do its work as well in Canada as in China, if it were not interfered with. And here doubt stept in. The pressure upon the War Department was urgent; the chief question appeared to be to throw into Canada the largest number of troops in the shortest space of time; the people were part of ourselves; they lacked nothing except trained soldiers and military stores. Would the old leaven of military prejudice come to the surface again, and the Medical Department be once more rendered comparatively helpless, by its requirements being made to give way to the more apparent military—that is to say, fighting efficiency of the troops?

We doubted. Whether it was the unfortunate march of the Guards to Guildford in the past summer which rendered us suspicious—a march which told of thoughtlessness or indifference among officers high in the service, and who had even not lacked experience in the field: whether it was that a report of stores being hurried pell-mell into one of the transports destined for Canada, awoke a fear that the Ordnance storekeepers had not yet acquired vulgar (sad to say), but straightforward and very necessary business habits, and revived the painful remembrance of that horrible time when, before Sebastopol, the doctors' hands were

---

* At a meeting of the Epidemiological Society, April 1st, 1861, the Director-General of the Army Medical Department made the following statement of the health of the troops engaged in the Chinese war from the 1st May to 11th Nov., 1860:—

| | No. | Deaths. | Mean sick. |
|---|---|---|---|
| Europeans . . | 9169 | 224 | 418 |
| Natives of India | 4920 | 79 | 156 |

| | Europeans. | Natives of India. |
|---|---|---|
| Ratio per 1000 of strength constantly sick . . . . . . . . . . | 46·09 | 31·71 |
| Annual ratio of deaths per 1000 of strength . . . . . . . . | 45·3 | 29·45 |
| Deaths exclusive of those killed in action or who died of their wounds | 38·43 | 27·96 |

tied, his help made unavailing, by the need of necessary medicaments and supplies, which, although in ships at anchor in the harbour of Balaklava, could not be got at or found, being hidden away among, or inaccessibly buried beneath, heavier, and for the time immovable, stores—stowed away, indeed, pell-mell. Nay, in the seeming indifference which led to a repetition of this unbusinesslike and discreditable want of care and order in sending stores on board the transport, we were reminded of that still more horrible time when, while our men, in the camps before Sebastopol, were languishing for a more varied diet; while they were becoming tainted, and subsequently when they were decimated, with scurvy; while the horrors of the awful winter of 1854-55 were aggravated for want of food more fitted for their wretched state; while they were tantalized in their misery by the green coffee served out to them, vast cargoes of rice, potatoes (preserved and dried), peas, Scotch barley, &c., and thousands of pounds of lime-juice and tea, were lying at Balaklava, unknown to the Commander-in-Chief and commanding officers generally, but known to the Commissariat, and to the officers of this department only. Whether it was the assertion that, notwithstanding the experience of the Crimea, no long boots for the soldiers were kept in store, and that France had helped us out of a difficulty occasioned by their sudden want for Canada, led us to fear that Army Sanitary Reform was not yet altogether sound at the core; whether it was that certain rheumatic pains, reliques of vigils, starvation, and exposure in the Sebastopol campaign, and which had crippled our sinister leg, had induced a tendency to carp at anything or everything connected with military movements; or whether it was that the gallant band of men hastening o'er the storm-tossed Atlantic to the deep snows and ice-locked boundary line of Canada, recalled so vividly as to enfeeble the remembrance of much that has happened since, the bitter memory of the tempest-vexed Euxine and the desolate heights and plains of the Chersonese, at that time when—

> Praying breath rose white in air,
> Eyes were set in a stern stare,
> Hands were stretcht for help that came not as they sank in silence low;
> Our grand, our gracious dead,
> Who lay down in their death-bed,
> With their winding-sheet and wreath of winter snow;

—whether it was one or all of these considerations which led us to doubt, we know not, but we doubted, and, happily, without just cause.

It is now known that no pains were spared to provide for the care of the men destined for Canada, whether sick or well; and there is good ground for the belief that the sanitary condition of the force in British America will redound as greatly to the honour of the Army Medical Department as the sanitary condition of the troops who passed through the Chinese War. The excellent requirements of the New Code of Medical Regulations it would appear have been carried out to the letter; the food and clothing of the troops have received fitting attention,—the daily ration of fresh meat having been increased, according to the direction of the home authorities, to one pound and a quarter, and instructions given to augment it to one pound and a half if needful; and each soldier has been provided with the following extra articles of clothing: two pairs of woollen drawers, two pairs of worsted stockings, two merino under-vests, one chamois-leather waistcoat, one Jersey, one pair of seal-skin mits, one seal-skin cap with ear-muffler, one sheep-skin overcoat, and one pair of Canadian boots; also one pair of fur and buffalo robes for the sleigh. Further, every care was taken for the welfare of the men over the route they traversed on landing. Huts, properly warmed and ventilated, were prepared for shelter and repose at intervals of thirty miles, and arrangements made for serving out hot coffee or spirits and water with the meals. Provision was also made for the sick, and the medical officers placed upon their guard as to the diseases most to be apprehended among the troops. So efficient, indeed, were all the arrangements made, that it is understood the ratio of sick to strength throughout both the sea and the land passage has not exceeded two per cent.

This is as it should be, and affords another striking illustration how greatly the forces of the nation may be economized, when the sanitary condition of the soldier is regulated by the teachings of both reason and experience. The military efficiency of an army is in fact mainly, and in the long run, entirely, dependent upon its sanitary condition. But neither the Government, nor the military authorities, nor Parliament, nor the people, would give due heed to this oft-iterated truth,* until it was driven closely home by the events of the Crimean war. In like manner, our civil population had persistently turned a deaf ear to the doctrine, that the wealth and prosperity of a nation were intimately linked to the health-condition of its people, until they were frightened into attention and belief by recurring pestilential outbreaks of cholera.

* "—— Thy chief anxiety," said Cambyses to Cyrus, speaking of the management of an army, "should be to provide for health; for thou oughtest to take care to prevent the army from falling into sickness at all." Cambyses laughed when his son told him that his military tutor had taught him only military tactics, and never mentioned "the domestic affairs of the army."—*Cyropædia, bk.* I.

The Crimean war constitutes the great epoch of sanitary reform, or rather revolution, in the army. This war will ever be as memorable for its sanitary as its military results. Commencing with a mortality among the troops, mainly occasioned by disease, which exceeded even that caused by the devastations of the most malignant pestilence in a civil population, it ended with a death-rate less than *half* that which existed in the army when stationed at home. The paradox was a strange one, but the one extreme served but to clench the truths to be gathered from the other. Throughout the seven months from October 1st, 1854, to April 30th, 1855, when the men were harassed by work altogether disproportioned to their strength, and by broken rest; when they were subjected to all the evils arising from insufficient clothing and shelter, and scanty and unwholesome food, in the wet and tempestuous wintry season, the rate of mortality rose in the army in the East to 600 per 1000 per annum. In January, 1855, the mortality ranged even to no less than 1,173½ per 1000 per annum; but in November and December, 1855, when supplies had long been regular and abundant, the food wholesome, and the camps subjected to stringent sanitary supervision and regulations, the death-rate per 1000 had fallen to 44 and 33; from January to May, 1856, to 12½; and in May the mortality fell even so low as 8 per 1000—the annual average of deaths, in the same proportion of men, in the Line, at home, being 18·7, and among the Guards 20·4.

The grief, the indignation, the alarm which swept over the country when the disastrous condition of our gallant troops in the winter of 1854-55 became known, happily compelled the Government to institute searching and immediate inquiries into the causes which had led to the horrible hardships that threatened at one time to annihilate the army in the field. Happily also these inquiries brought clearly and indisputably to light the sad fact, that these hardships were not the unavoidable contingencies of a winter campaign in a country far separated from needful supplies, but that they were the natural, but exaggerated, reflex of the ordinary state of things—of the ordinary government and management of the army at home.

The feelings indignantly revolt even now, when we recall to mind the thoughtlessness, the indifference, the ignorance, the wilfulness which played so prominent a part in bringing about the disasters of that terrible winter; which were responsible for the perpetuation of a senseless routine that set at nought the teachings of both reason and experience; and which effectually manacled the many able, and willing, and anxious hands in the service who would otherwise have helped most effectually.

In estimating the indifference and wilful blindness which led to the disasters in the Crimea, it is not to be forgotten that the salvation of the army was ultimately effected by measures neither new nor unfamiliar in military annals.

The experience of previous wars, as abundantly recorded in the writings of Sir John Pringle, Sir James McGrigor, Dr. Hennan, Mr. Guthrie, and others, had taught all those great truths respecting the welfare of an army in the field, which had to be re-learned from the terrible lesson before Sebastopol. But although these truths were familiar to military medical men, and had often been brought home to the Horse-Guards and the Executive by calamities in camp and in hospital, they were never permitted to have that weight with the authorities (particularly the military authorities) which would have led to their sound practical application. Hence the expedition to the Crimea, in all that related to the sanitary care of the troops, was scarcely better provided for than the first expedition to the Peninsula, half a century ago.

Again, it was no new fact to find troops in better health during a campaign than when stationed at home. The annual ratio of mortality per 1000 among the Guards who served in Canada from 1838 to 1842, amounted to 14·5 ; while at home in the ten years 1837—1846, the death-rate among the Foot Guards was no less than 20·4. The mortality among the Infantry of the Line serving in Canada during the insurrection, differed, moreover, but a fraction from that experienced among the same class of troops at home.

Further, the importance, nay, the necessity, of an accurate knowledge of the sanitary condition of the army in the maintenance of its efficiency, had been most fully shown from time to time. Five statistical reports on the health of the army at home and abroad, were presented to the Government, at irregular intervals, from 1838 to 1853 inclusive. Two illustrations will suffice to indicate the important results which followed from the facts made known in, and the suggestions adopted from, these reports. During the twenty years prior to 1838, the annual mortality among the troops in Jamaica had averaged 140 per 1000 of the strength ; this ratio has been reduced so much, that within the last four or five years the mortality has only averaged 30 per 1000. In like manner the mortality among the troops in Ceylon, which aforetime averaged 75 per 1000, has been reduced to 38 per 1000.

The sanitary teachings of peace, however, failed, equally with those of war, in inducing the Government and the War-Office to give that systematic and just attention to the subject which was demanded by its importance, until the awful calamity of 1854-55 came upon us.

The most momentous result of the Russian war was undoubtedly the Royal Commission appointed to inquire into the regulations affecting the sanitary condition of the army ; and which brought clearly to light the fact, that the disasters in the Crimea were a natural sequence of our then military administration.

It was certainly somewhat astounding to learn, as we then did (although not for the first time by those who were familiar with existing army statistics), that the mortality in the army was double that found among civilians at army ages. Such, however, was the fact, as the following figures show:

*Rates of Mortality per* 1000 *per annum.*

| | |
|---|---|
| Effective men of all ages of the Army at home: | |
| Total | 17·5 |
| Household Cavalry | 11·0 |
| Dragoon Guards and Dragoons | 13·3 |
| Foot Guards | 20·4 |
| Infantry of the Line | 18·7 |
| Population of England and Wales—army ages: | |
| Town and country population | 9·2 |
| Country alone | 7·7 |
| One of the unhealthiest towns—army ages: | |
| Manchester | 12·4 |

*Rates of Mortality per* 1000 *men of the Army at home, and of the English civil Male population, at corresponding quinquennial periods, as stated by the Registrar-General.*

| | | |
|---|---|---|
| Ages, 20 to 25 | —Civilians | 8·4 |
| " " | Soldiers | 17·0 |
| " 25 to 30 | —Civilians | 9·2 |
| " " | Soldiers | 18·3 |
| " 30 to 35 | —Civilians | 10·2 |
| " " | Soldiers | 18·4 |
| " 35 to 40 | —Civilians | 11·6 |
| " " | Soldiers | 19·3 |

"From this," the Report of the Commission observes, "it appears that if the army at home were as healthy as the population from which it is drawn, soldiers would die at one half the rate at which they die now."

Such a result was pretty nigh incomprehensible when it was considered that the soldiers were picked men, and that they were seemingly removed from all those disadvantages which affect the health of the labouring classes in ordinary life. Further examination, however, showed that this anomalous result was to be assigned to two categories of causes, to wit, (1) want of exercise and suitable employment among the troops; and (2) crowding and insufficient ventilation, and nuisances arising from latrines and defective sewerage in barracks.

To the want of due exercise might justly be attributed some portion of the insanitary condition of the army, consistently with what had been observed of the influence of the same cause in civil life. Moreover, it was reasonable to conclude that the different rates of mortality observed in the Cavalry of the Line (13·6 per annum), and in the Infantry of the Line (17·9), might be assigned in some measure to the greater amount and variety of exercise undergone by the former. The trooper, in fact, passes more time out of doors than the foot soldier, his duties being partly on horseback and partly on foot; and the need of grooming his horse, as well as his sword exercise, call into action a different set of muscles to those used in marching and in infantry drill, and require more varied action. "Indeed, all the attitudes of the foot soldier on parade, at drill, and on the march, are singularly monotonous and constrained, and it is rare to see an infantry soldier whose figure is as fully developed or as well set up as that of a cavalry soldier."* The low rate of mortality in the navy (8·8 on the home-station), in which the men, though necessarily berthed in a very confined space, undergo an immense amount of exercise, calling the greatest variety of muscle into play, and pass a large proportion both day and night in the open air, appears to favour the opinion above expressed.

But while assigning just weight to the influence of insufficient exercise in depressing the health of the army, it was unquestionable that the great, the most efficient, cause of its insanitary condition, was the foulness of the soldiers' residences, and the vitiated state of the atmosphere in which the men were compelled to pass the greater portion of their life. The predominance of pulmonary affections among the causes of death in the army was highly significant, as bearing upon the influence of a foul atmosphere upon the health of the men. While in civil life, at army ages, the deaths by pulmonary diseases were 6·3 per 1000, in the Cavalry they amounted to 7·3, in the Infantry of the Line to 10·2, and in the Guards to 13·8; and of the entire number of deaths from all causes in the army, diseases of the lungs constituted no less than 53·9 per cent. in the Cavalry, 57·27 per cent. in the Infantry, and 67·68 per cent. in the Guards.

It could not be alleged that, as is frequently the case in civil life, the clothing, the food, and the nature of the occupation itself were of such a character in the army as to be predisposing causes of the excessive mortality of the soldier from pulmonary disease. Now, if it could be shown that the soldier in barracks breathes a vitiated and polluted atmosphere, it would follow that of the four causes chiefly predisposing to pulmonary affections, namely, insufficient clothing, insufficient and unwholesome food, nature of occupation, and foul atmosphere, the last is the one to which the excessive liability of the soldier to this class of diseases

* *Report*, p. xiv.

must be assigned. Many of the maladies included under the general head of chest and tubercular diseases cannot, however, be attributed directly to breathing a vitiated atmosphere. But, excluding inflammation of the lungs and pleura, acute catarrh, and some other diseases which may have no obvious connexion with the purity or impurity of the atmosphere, it was found that consumption, chronic catarrh,* spitting of blood, asthma, and difficulty of breathing, alone occasioned a mortality of 12·5 per 1000 mean strength in the Guards and 8·9 in the Infantry of the Line.

The extent to which our soldiers were exposed to a vitiated and polluted atmosphere was made sufficiently manifest in the evidence laid before the Commission on the Sanitary State of Barracks. From this it appeared that the dormitories or barrack-rooms were very confined; that although the minimum cubic space allowed to each soldier by regulation was only 450 feet, in the majority of cases this minimum was not attained; that the regulated space of one foot between the beds was also practically unattained,—yet it was certain that the amount of interval allowed between the beds is of more consequence, so far as health is concerned, than the mere amount of cubic space given above by the altitude of the room; that owing to the ordinary construction of the buildings, barrack-rooms rarely have windows at opposite sides or ends of the room, and there are, consequently, very insufficient means of ventilation; that where ventilators existed, "they were frequently stopped up by the men themselves, who come from a class very little persuaded of the advantages of ventilation, and whom poverty has accustomed, from their youth up, to look to the exclusion of the external air, in the absence of fuel, as the best mode of securing warmth;" that "barrack-rooms are occasionally found in the basement of the building, approached by descending steps from the natural surface level, the tops of the windows, which open on one side only of the rooms, being little, if at all, above such surface level; and in low rooms thus situated men may be found lodged in beds so closely ranged that the side of one touches the side of the other;" that the result was, "that the soldier sleeps in a fetid and unwholesome atmosphere, the habitual breathing of which, though producing, for the most part, no distinct immediate effects, probably lays the seeds of that pulmonary disease which is so fatal in the British Army:" of this atmosphere "abundant evidence, though of a most disgusting nature," was laid before the Commission; that, finally, the latrines, cesspools, and drains were most defective, and that to this source "is probably to be attributed much of the excess of

---

* Most of the deaths so recorded were in reality consumption.—See *Statistical Report of Army,* 1853, p. 20.

mortality by fever which the army statistics show over that arising from the same class of diseases in civil life; their respective rates being: Deaths by fever in civil population in towns, 1·2 per 1000; in the Cavalry, 1·4; in the Guards, 2·4; in the Infantry, 2·5; and in the Artillery, 1·9,—for the ten years ending 31st March, 1857."

Very shortly after the Commission on the Sanitary Regulations of the Army had closed its sittings, a Commission was appointed to inquire into the Sanitary Condition and Improvement of Barracks and Hospitals. The Report of this Commission, published in the course of the past year, confirms, in every respect, the conclusions of the first-named Commission on the sanitary state of barracks. The Barrack and Hospital Commission was authorized to direct the immediate execution of such works as might appear to it to be necessary for the ventilation, warming, lighting, draining, and sewering of, and the securing a sufficient supply of good water for, both barracks and hospitals, provided the cost of the works did not exceed 100*l.* for each hospital or barrack where such improvements were needed. But the Report states that this sum was "found to be totally inadequate even for the execution of the most urgent sanitary works," since, "although large sums of money have from time to time been spent on these barracks and hospitals, a very small proportion of it appears to have been devoted to sanitary purposes. So far, indeed," it is added, "as concerns the health of the troops, almost every barrack and hospital we have visited can be considered in no other light than as never having been completed;" and the funds required for the necessary improvements have consequently exceeded in amount what could have been anticipated when the Commission entered upon its work. (p. 10.)

A few illustrations from this Report may be cited with advantage:—

As might have been anticipated from the death-rates already quoted, the sanitary condition and construction of the Guards' barracks in town were found to be most defective. The interior of the buildings presented a majority of the evils already referred to, not, perhaps, more conspicuously than in many other barracks; but we refer to them for the sake of an additional, and most striking example of the comparative health of troops in barracks and in camp.

The Guards are, perhaps, more continuously barracked in town districts than any other part of the army, and if we compare their mortality in the London barracks, and also the relative proportion of admissions from diseases of the zymotic class, with the death-rate and sickness at Aldershott and Shorncliffe, we find the following results:—

| | Deaths per 1000 per annum. | Admissions per 1000 per annum. | | |
|---|---|---|---|---|
| | | Fevers. | Diarrhœa. | Total. |
| Guards—1847-54. . . . | 15·24. | 51·86 | 61·32 | 113·18 |
| Aldershott / Shorncliffe } 3 years ending Dec. 31, 1859 | 4·7 | 37·5 | 17·5 | 55.0 |

From these figures it would appear that while the Guards were in camp the amount of sickness was reduced one-half, the mortality two-thirds. To estimate the value of these results it is needful to bear in mind that the number of men barracked in town districts in the United Kingdom amounts to upwards of 25,000. Hence, among other suggestions, the Commission recommends that, whenever military requirements will permit it, barracks should be placed in the open country, or in the open suburbs of towns.

The defects of barrack construction were found to be multifarious, and appear to have arisen from the simple fact that buildings were designed (with few exceptions) without any reference to the health of the inmates, but with the object of getting as many men as possible housed in the smallest space.

Although the minimum cubic space allowed to each soldier in permanent barracks, by the old regulations, was 450 feet, the Commission ascertained that no less than 1335 men, equal to one regiment and a half, were living and sleeping in rooms with less than 250 cubic feet per man; 15,195 with less than 350 feet; and 34,882 with less than 400. Moreover, 65,271 men had less than 500 cubic feet per man, and of the whole force, 76,813 men, only 4656 had sleeping room exceeding 550 cubic feet each, and of these only 2003 had an allowance of 600 cubic feet per man. The present regulations provide that the minimum cubic space per man shall be 600 feet. The overcrowding of barracks may be further estimated by the fact, that the deficiency of barrack accommodation, at different stations in Great Britain and Ireland, varies from 21·6 per cent. (Woolwich) to 43·4 per cent. (Chatham).

The Report further shows that there was "a total want of any proper systematic method of ventilation," and that the gravest defects also existed, with few exceptions, in the arrangements for warming and cleansing, in the water supply, drainage, cookhouses, ablution-rooms, wash-houses, guard-rooms, &c.

The chief results of the Commission on the Sanitary Regulations and State of the Army are aptly summed up in a letter of the late lamented Lord Herbert, prefixed to the New Code of Medical Regulations for the Army. He writes:—

"The Report of the Royal Commission has shown the high rate of mortality from disease existing among the troops at all times, but more especially during war—the defective condition of military hospitals—*the absence of any means by which the improvements recently introduced for the protection of health, in civil life, can be rendered available in Barracks, Camps, and Hospitals* [the chief clue to, and chief connecting link between, the sanitary disasters in the Crimea and the high rate of mortality among the troops at home],—and the great loss of life arising from these defects during the late war with Russia.

"The existing regulations," Lord Herbert continues, "appeared to us by no means sufficient to prevent the recurrence of such losses of life and efficiency in the army. On some most important points they are altogether silent.

"The duties to be performed are not sufficiently defined, nor are the responsibilities clearly placed on those charged with specific duties; and, even should a medical officer discern his duty and perform it, there is no necessary connexion between any recommendations he may make, and their being carried into effect.

"To obviate these evils as far as practicable, the Royal Commission recommended two fundamental changes in the Army Medical Department; and unless they be carried out, any attempt at improving the existing regulations will be of little avail. One of these changes is the remodelling of the department; the other is, the organization of a practical Army Medical School."

These changes have now been happily effected. Heretofore it rested with the Director-General to fulfil *all* the requirements, administrative and scientific, of his department. A task manifestly beyond at least the physical capabilities of any one man. There was no one attached to the department specially conversant with army hygiène or sanitary science; no office in which the Army Medical Statistics—the very basis of all scientific procedure and the gauge of sickness or health of the troops—could be reduced; no means of rendering available for the public service the large amount of information and experience on Army diseases which is constantly being accumulated in the Department. Under the recommendations of the Royal Commission the Director-General's Office has been subdivided into three distinct branches, medical, sanitary, and statistical, each under a separate head, who should work the routine details of his department. To the heads of these branches are referred questions connected with the medical and sanitary duties and statistics of the army, and the chiefs of the three branches constitute a consultative council to assist the Director-General with their advice on subjects coming within their respective branches,—the whole proceedings of the department, nevertheless, going on through the Director-General, and under his direction, as the sole responsible administrative head.

The Army Medical School is now in full operation, and in it the Army Medical Man, at the outset of his career, receives a very necessary specific training in the special sanitary and medical duties he is required to perform, in accordance with the New Code of Regulations for his guidance. These regulations, in addition to the modifications in the Medical Department, already referred to, remedy more or less effectually the defects previously existing in the hospital and sanitary administration of the army,—much prominence being given to Army hygiène, "because," as Lord Herbert remarks, in the letter already referred to, "it must now be considered as a matter of primary importance to the public interests that every resource of modern science should be called into requisition for preserving the health and physical efficiency of the Army."

To give effectiveness to the whole sanitary administration of the Army, the New Regulations require that periodical statistical and sanitary returns (weekly, monthly, quarterly, and annual) shall be sent by medical officers to the Director-General. These returns will keep the Director-General accurately acquainted with the health of the troops in all parts of the world, and a summary of their results will henceforth be accessible to the public in the Report on the Health of the Army, which the Director-General is now required to present annually to the Secretary of State for War, and which is laid before Parliament.

The first Annual Report of the Director-General* was presented to Parliament in the course of the last Session, and from it we obtain a most hopeful idea of the prospective sanitary welfare of the soldier. This will be made evident by the following table, showing the rate of mortality (corrected according to the proportion of men serving at different periods of life) among the troops serving in the United Kingdom in 1859, in comparison with the mortality of the ten years 1837-46 :—

| | Annual Ratio per 1000. | |
|---|---|---|
| | 1859. | 1837-46. |
| Household Cavalry | 4·27 | 10·39 |
| Dragoon Guards and Dragoons | 7·94 | 12·08 |
| Military Train | 7·09 | |
| Foot Guards | 8·59 | 18·72 |
| Infantry Regiments | 7·58 | 17·06 |
| Depôt Battalions | 12·26 | |
| *Civil Population in Healthy Districts* | 7·23 | |

This table, as Dr. Balfour, the able head of the statistical branch of the Army Medical Department, justly observes, "shows a remarkable and most satisfactory reduction in the amount of mortality in all classes of troops ; indeed, except in the Depôt

* *Army Medical Department.—Statistical, Sanitary, and Medical Reports for the year* 1859, p. 438.

Battalions, it is little above that of the civil population in the healthiest districts of England." He adds:—

"The Foot Guards can scarcely be held as on an equality in a sanitary point of view with the inhabitants of such districts, being quartered chiefly in London, and with some of their barracks in the most densely populated quarters; but even among them the mortality is lower than among the male population at the same ages throughout England and Wales. In the case of the Household Cavalry the numbers are so limited that the proportion might be expected to fluctuate considerably; but in the others the marked decrease can hardly be deemed accidental. It cannot be attributed to the contingency of an unusually healthy season, for the mortality of the civil population, in 1859, did not differ materially from the average of former years. Nor has it arisen from a greater proportion of men being discharged as invalids, for, as will hereafter be shown, the number thus disposed of was, except in the Foot Guards, under the previous average. It may, perhaps, have been in some degree the effect of selection, as so much larger a proportion of the force than formerly consisted of men recently enlisted, and who may not have been exposed for a sufficient length of time to the deteriorating influences which affect a soldier, to have materially suffered in health from them. It will, therefore, be interesting to observe, whether under improved sanitary conditions this diminished rate of mortality can be in future maintained." (p. 7.)

Much probably of the great reduction of mortality in 1859 may have been the result of sanitary improvements, which had, even up to that time, been introduced into barracks and hospitals,* and which are still being carried out, more especially in respect to ventilation, drainage, food and cooking, according to the suggestions of the two Royal Commissions of 1857. In the course of 1859, the new system of ventilation introduced by the Barrack and Hospital Commission had been extended to the principal barracks and military hospitals of the United Kingdom; much had been done to amend latrines, urinals, and sewerage, and great improvements had been effected in the means of cooking. But it was not until after the publication of the New Code of Regulations in October, 1859, that the special sanitary duties of medical officers, instituted by the Regulations, were entered upon. Moreover, the funds disposable for sanitary improvements are limited, and, as a consequence, some little time must elapse before the whole of the suggestions of the Barrack Commission can be carried into operation. Hence it is reasonable to hope that when the sani-

* "There are numerous circumstances which have led to this reduction; but I believe that a considerable portion of it is due to the sanitary improvements consequent upon the statistical results which were brought out by our investigation" (referring to the Royal Commission on the Sanitary Condition of the Army in 1857, of which Dr. Balfour was Secretary).—*Dr. Balfour on the Vital Statistics of the Army. Report of International Statistical Congress,* 1860, p. 363.

tary measures now in progress of execution have been perfected, and the sanitary regulations of the New Code have been some time fully in action, the health of the Army will reach a still higher pitch than it did in 1859.

The subjoined table, compiled from the Statistical Report for 1859, shows the annual ratio of sickness and death in the principal military stations in 1859 and certain previous years:—

| | Ratio per 1000. | | | |
|---|---|---|---|---|
| | 1859. | | 1837—46. | |
| | Sickness. | Death. | Sickness. | Death. |
| Gibraltar | 949 | 7·18 | 939 | 13·58 |
| Malta | 1214 | 19·02 | 1120 | 16·77 |
| Ionian Islands | 881 | 11·75 | 1138·7 | 17·94 |
| Bermuda | 537 | 13·95 | 1334 | 33·8 |
| Nova Scotia and New Brunswick | 557·8 | 7·22 | 900·3 | 16·0 |
| Canada | 545 | 10·42 | 981·7 | 17·42 |
| Newfoundland | 1330·1 | 4·8 | 781 | 11·54 |
| Windward and Leeward Command: | | | | |
| *White Troops* | 1128 | 19·75 | 1936 | 69·54 |
| *Black Troops* | 1012·7 | 16·66 | 768 | 32·16 |
| | | | 1817—36. | |
| Jamaica: | | | | |
| *White Troops* | 1335 | 14·42 | | range from 61 to 307 |
| | | | 1840—49. | |
| *Black Troops* | 1281 | 30·95 | 834 | 29·86 |
| | | | 1817—36. | |
| Bahamas* | 832 | 40·3 | 1070 | 40·1 |
| | | | 1822—36. | |
| Honduras* | 850·8 | 6·2 | 881 | 30. |
| Western Africa†: | | | 1819—36. | |
| *Sierra Leone* | 542 | 14·02 | 812 | 30·1 |
| *Gambia* | 653 | 25·4 | | |
| *Gold Coast* | 581 | 25·06 | | |
| | | | 1837—46. | |
| St. Helena | 802 | 12·90 | 943 | 16·62 |
| Cape of Good Hope | 967 | 12·22 | 945 | 16·54‡ |
| Mauritius | 1237 | 16·04 | 909·6 | 22·38 |
| Ceylon: | | | | |
| *White Troops* | 1693·3 | 35·06 | 1444·2 | 41·74 |
| *Black Troops* | 724·4 | 10·19 | 1010·9 | 26·71 |
| Australia | 661·5 | 10·1 | | |
| | | | 1844—56. | |
| Tasmania | 416·1 | 15· | 726 | 11·87 |
| New Zealand | 636·4 | 4·5 | 529 | 12·8 |
| China: | | | | |
| *White Troops* | 2783·2 | 59·35 | | |
| *Native Troops* | 2783·2 | 41·93 | | |

* Black troops chiefly. † Black troops.

‡ Exclusive of killed in action. If the losses of the 59th Regt., broken down from disease in China, and sent to the Cape for change of climate, be added to the ratio of sickness and death for 1859, the figures amount to 1·077 and 14·57.

It is gratifying to learn from these figures that the reduction of mortality and increased health of the Army in 1859 was not confined to England only, but extended also to the majority of foreign stations. Where, moreover, an excess of sickness and mortality was noted, and where, indeed, there was an exaggeration of some one disease, it was chiefly in connexion with manifest and remediable sanitary defects, such as over-crowding and foul and noxious effluvia, to which the troops were exposed. Thus, an increase of fevers at Gibraltar is assigned to over-crowding of barracks; and prevalence of fever at Malta is attributed also to the same cause, as well as to the quality of the water in the tanks in the dry season, and local defects in certain barracks, including imperfect sewerage, or exposure to sewage air.

Rightly to appreciate the nature of the sanitary measures now in progress of execution or suggested for the welfare of the Army, it is necessary to turn to the Report of the Barrack and Hospital Commission. This Report, and the Report of the Sanitary Commission despatched to the Army in the Crimea, should be in the hands, or placed within reach of every Army medical officer, as two of the most complete, practical, and instructive studies of camp, barrack, and hospital hygiène existing. The Report of the Barrack Commission has, however, a much wider interest than that which arises from its special subject alone. For the Report deals with and throws light upon many important questions of common interest to all who are concerned in the Public Health.

To the solution of one of these questions only can we devote space, but this single illustration will suffice to show the great interest and importance of the Report.

One of the most difficult problems presented to the consideration of the Commission was the ventilation of barracks. The problem requiring to be solved is thus stated:—

"*In a building consisting of a number of rooms, generally entered from a common passage or staircase, sometimes directly from the outer air, and each having an open fireplace, which it is essential in every instance to retain, how to supply at all seasons and temperatures, and by day and night, each room by itself, and independently of every other room, with a sufficiency of air to keep the room healthy, and at the same time to prevent the temperature from falling below what is required for the comfort of the men. To do this with the least possible interference with the structure of the rooms, on a plan not easily deranged, and at a minimum cost?*"

The construction of the buildings and the retention of an open fireplace set aside the possibility of adopting satisfactorily any artificial system of ventilation in use, whether by propulsion or extraction—the former objection preventing the application of any of these systems on a uniform principle, the latter being incompatible with artificial ventilation. Certainly, air might be driven into a room with a fire in the grate, but then the advantages of the artificial method are lost, and the room can be ventilated much better without it. Again,

"The open fireplace is, if possible, less adapted for rooms ventilated by extraction than for rooms ventilated by propulsion. The chimney, with its fire, is in itself a powerful extracting shaft; but if the extracting shaft acted as it ought, with a predominating power, it would draw the smoke down all the chimneys. If, on the other hand, the chimney-draught were the strongest, air would be drawn down the extraction shaft."

The retention of the fireplace and the construction of the buildings were also fatal objections to the adoption of Watson's, Mackinnel's, or Muir's self-acting ventilators.* The double action of these ventilators is destroyed, and they become incast shafts only, by the action of a fireplace with fire in it; and the same result follows from the opening of doors or windows. They are chiefly adapted, indeed, for single rooms standing apart, such as churches, chapels, schools, libraries, &c., warmed by stoves, and where the doors are shut for hours at a time. Mr. Watson's plan was adapted, under his own supervision, to one of the blocks of houses at the Wellington Barracks, containing twelve rooms.

"He introduced," the Report states, "his ventilator at the top of the staircase which passes up the middle of the house, and inserted louvers in the partition wall between the staircase and each of the twelve barrack-rooms. It was anticipated that an air current would descend through one division of the tube into the staircase, would pass thence through one set of louvers into each barrack-room, would

* These ingenious ventilators require fixed conditions for their perfect action. Alter these conditions, and they become wholly outlet or wholly inlet. "The condition essential to their operation," says the Report, "is that the room to which they are applied be closed, and in a closed room their action is singular. If a number of people be crowded into a room with the fireplace, doors, and windows shut, and if a tube of an apparently sufficient area to afford ventilation for the inmates be carried from the ceiling of the room above the roof of the building, there will be an irregular effort at effecting an interchange between the air of the room and the outside air. The outer air will descend, and the inner air will ascend in fitful, variable, irregular currents, and the room will be badly ventilated, if ventilated at all. But singularly enough, no sooner is the tube divided longitudinally from top to bottom by means of any division, however thin, than its action becomes immediately changed, a current of air descends into the room continuously on one side of the partition, and a current of foul air ascends from the room continuously on the other side of the partition. One-half the tube supplies fresh air to the inmates of the room, and the other half removes foul air, so that if the size be properly adjusted, the air in the room is kept sweet." (p. 69.)

return by the other set of louvers into the staircase, and pass up through the second division of his ventilator, and so escape. On examining the operation of the apparatus, however, it was found that the current in both divisions of the ventilator passed down into the staircase through both sets of louvers into the rooms, and thence up the chimneys, so that there was no up-current in the ventilator at all." (p. 70.)

Dr. Arnott's Chimney Valve and Sherrington's Ventilator (an inlet for fresh air placed close to the ceiling of the room) were found economical and efficient ventilating agents in rooms not occupied by several people, such as non-commissioned officers' rooms, and were recommended to be adopted under these circumstances by the Commission, but neither plan of ventilation is fitted for rooms in which a number of persons live together.

None of the plans of ventilation investigated or submitted to the Commission meeting the requirements of the problem to be solved, the Commission set itself to study the question practically.

And first, it was endeavoured to be ascertained approximately what was the amount of fresh air required per man. Existing estimates of this amount were very various. Chemistry teaches us that at least 200 cubic feet of air per hour is required to dilute the carbonic acid and water given off from the body to the same standard as they exist in the atmosphere.

"But," says the Report, "chemistry takes no cognizance of those aerial poisons eliminated from the skin and lungs, and which in stagnant air have been diluted to the extent stated. Indeed, the object to be served by ventilation is primarily the dilution and removal of these poisonous exhalations, and if this be secured the carbonic acid and water will be removed at the same time.

"Few persons," the Report adds, "are, perhaps, aware that an ordinary barrack fireplace removes a much larger amount of air than is required merely to dilute the carbonic acid and water to a healthy standard. The quantity varies, of course, with the section, height, and temperature of the chimney-flue, and also with the force and direction of the wind. The extremes may be practically assumed at from 6000 cubic feet per hour up to ten times that amount. A twelve-man room, affording 500 cubic feet per man, would on the lowest estimate have 500 cubic feet of air per man per hour supplied to it by the chimney-draught alone; that is to say, the firegrate will ensure a ventilation above twice as great as will fulfil the requirements of chemistry; and yet it has been ascertained by sufficient experience that rooms so ventilated are both offensive and unwholesome." (p. 72.)

This result is, no doubt, partly, if not chiefly, attributable to the fact that the chimney-draught is supplied mainly from the lower stratum of air in the room, the upper portion of the room forming, as it were, a reservoir of foul air situated above the ventilating power.

"The sense of smell," the Report continues, "affording the chief indication of the healthiness or unhealthiness of a room atmosphere, and differing as the delicacy of this sense does in different individuals, it is not perhaps possible to arrive at an absolute standard of ventilation; but in order to obtain some practical estimate of the quantity of air required to ensure this amount, we had air shafts, having certain definite sections, carried from the corners of the ceilings of twelve barrack-rooms in the Wellington Barracks, up through the roof, so arranged that the apertures might be contracted, and the quantity of air passing up each shaft measured by a delicate anemometer constructed by Naumann, of Paris, for the express object. The measurements were taken at different periods, during several months, between two and five o'clock in the morning. The requisite observations of temperature without and within the rooms, and of the hygrometric state of the air, were also taken, and the sensible state of the room atmosphere was observed at the same time. From these observations, as well as from others which we have been enabled to make, we are of opinion that an estimate on which we based our first improvement in ventilation, is sufficiently near the truth for practical purposes. It is as follows:—that in a barrack-room containing a number of men, at 600 cubic feet per man, the whole air of the room should be renewed at least twice in the hour. In other words, that each man should have in round numbers 1200 cubic feet of fresh air supplied to him per hour. Even this amount may not be sufficient to preserve a barrack-room entirely free of odour at all times and all seasons; but the difficulties of a thorough solution of a problem, where the conditions are so variable, have led us to adopt this as our unit of ventilation, while in the ventilating plans we have carried out, it is always possible to increase the amount without difficulty. After our plans had been for some time in operation, we were glad to learn, from a report on the warming and ventilation of dwellings, made to the General Board of Health, by Messrs. Fairbairn, Glaisher, and Wheatstone, that a similar unit, namely, from 15 to 20 cubic feet per man per minute, had been arrived at by these gentlemen. But while adopting this unit, we hold it at the same time to be an indispensable condition, that each man should have the amount of space, 600 cubic feet, recommended by the Royal Commission.

"But to ventilate a barrack-room, it is not only necessary to supply this amount of air, but to supply it at different seasons, during hot weather, during cold weather, and during what may be considered as the temperate days and nights of the year. During mild weather the problem is one of comparative facility. During warm weather, especially if the weather be at the same time moist, nothing short of open windows will keep a room comfortable in which a number of people sleep. This, indeed, is generally done by the soldiers for their own comfort. During cold weather, however, it is essentially necessary to provide for warming part of the admitted air." (p. 72.)

To meet the requirements not met by windows, the Commission determined to adopt a system of ventilation by which each barrack-room should be kept independent of every other room in this respect, and to depend for the movement of the air in the rooms "upon the fireplace and upon the element of the difference of temperature between the air outside and the air within."

The Commission recommends, therefore, that in each room a shaft of certain given dimensions, and having a sectional area adjusted to its length and to the number of inmates in the room (or when the number of occupants is governed by the cubic space, as it is to be hoped will henceforth be the case), to the cubic contents of the room, shall be carried from an angle of the ceiling three or four feet above the roof of the building. "The velocity of the air in the shaft, and hence its ventilating power, will depend, 1st, on the difference of temperature between the inner and outer air; 2nd, on the length of the shaft; 3rd, on the amount of friction in the shaft; and 4th, on the freedom, or otherwise, with which the air to supply the shaft enters the room." Shafts with a sectional area of one inch to every 50 cubic feet of room space are recommended for the top floor of a barrack; of one inch to every 55 cubic feet of room space for the floor next below; and one inch to 60 cubic feet of room space for the still lower floor, if the barrack consists of three stories.

"The velocity in these shafts," the Report states, "is dependent, of course, on the difference of temperature between the air in the room and the air without, on the amount of movement in the outer atmosphere, and other circumstances. When the temperature is nearly equal, as, for instance, when the windows are open, there is very little upward draught, except as the result of movements in the atmosphere without, but when windows are open the room is being ventilated without the shaft. At other times the current is energetic. From a number of observations made with Naumann's anemometer, we have found that in rooms in the Wellington Barracks, with a cubic capacity of 7920 feet, a quantity of air equivalent to from 8000 to 9000 cubic feet per hour passes up the shafts. Each shaft would, therefore, remove from the room about 600 cubic feet per man per hour, if the rooms were occupied by 13 men each, which is the largest number they ought to contain. We have thus obtained outlets for foul air capable of removing 600 cubic feet of air per man per hour; we have already seen that the chimney removes about the same quantity, and thus the amount of 1200 cubic feet is obtained. The amount of air varies so much that it is necessary to provide regulating valves, not under the control of the men, for the inlets; but these valves should never admit of being completely closed." (p. 73.)

The foul-air shafts already introduced into barracks have been made of three-quarter-inch deal, very smooth inside, and rebated and grooved together at the angles, but it would be better to have the shafts constructed of glazed pipe built in the wall, or to have smooth cement sides. The shafts require to be louvered at the summit to keep out wind and rain; it is requisite also to place inverted louvres at the lower end, to cast occasional down-draughts up to the ceiling.

If, however, a room has no other means of ventilation than a foul-air shaft and chimney-flue, the fireplace will supply itself at the expense of a down-draught through the shaft. It is necessary then to provide proper inlets for air, to supply both the fire and ventilating shaft. The nature, position, and dimensions of these inlets constitute the next question with which the Commission deals. Inlets situated near the floor are productive of great discomfort, by reducing the temperature of the lower stratum of air and causing cold currents. As a consequence they are almost invariably tampered with, to the great and often serious detriment of the ventilation of a room. The Commission ascertained by actual experiment, that air admitted near the ceiling of the room soon ceased to be detected as a distinct current, and that at a short distance from the inlet it had mingled with the general mass of the air and had disappeared. It was decided then "for practical reasons, fully sustained by the results of experiment," that inlets for air are best placed close to the ceiling. The form of inlet recommended and adopted is that of iron or perforated air-bricks of different sectional areas—1 square inch being allowed for every 60 cubic feet of contents of room, or 1 square inch to every 120 cubic feet if warm air is admitted round the fire-grate. Two inlets are recommended for barrack-rooms of ordinary size, one to be placed on each of the opposite sides of the room, but not opposite each other, or both on the same side if the rooms be back-to-back. In larger rooms the number of inlets is to be increased.

In order to prevent draughts, as far as practicable, the inlets should be covered by a wooden cornice several times their length, and sloping upwards to the ceiling at an angle of 45°. The upper side of the cornice is to be formed of perforated zinc with holes of $\frac{1}{6}$ to $\frac{1}{8}$ of an inch diameter. The front of the cornice opposite the inlet should be of wood, to break still further the force of the current. The area of perforated zinc, through which the air passes into the room, is from six to eight times the area of the inlet from the outer air. The accompanying diagrams (Figs. 1 and 2) show the elevation of the ventilating cornice over the inlet, and a section of the inlet.

FIG. 1.—Elevation of ventilating cornice over inlet.

Several separate small inlets terminating externally in an ordinary air-brick, internally in louvres sloping up to the ceiling, and capable of being closed at pleasure, would be better in new buildings; or Sherringham's ventilator would be applicable.

FIG. 2.—Section of inlet, showing the perforated zinc cover within the room, and the arrangement for closing the inlet with a valve and cord working on pivots fixed to its lower edge, and so adjusted that, by being weighted on its upper edge, it will fall down and leave the inlet open when not purposely raised and held up by a cord to close it. The valve should fit very loosely, so as to leave, when closed, at least from half an inch to one inch between it and the sides and bottom of the inlet hole. The valve to be made of zinc or galvanized iron. A, ceiling; B, external wall; C, iron grating; D, valve.

The relative position and arrangement adopted for the outlet shafts and inlets are shown in the following diagrams (Figs. 3 and 4). It is important that the shafts and inlets should be placed as far from each other as possible, in order to secure the most thorough diffusion of the inflowing fresh air.

The large amount of air passing through a barrack-room in winter, with this plan of ventilation in action, would keep the room at a comparatively low temperature, unless some simple method could be adopted for warming a portion, at least, of the air admitted. To meet this requirement the Commission has suggested and carried out the following ingenious expedient,

FIG 3.—A, line of ceiling; B, upper room; C, lower room; D, inlet; E, shaft from lower room; F, shaft from upper room.

FIG. 4—Half plan of a barrack-room. A, outlet shaft; B, inlet.

effected by remodelling the fire-grates used in barracks. The new grates recommended are constructed as follows:—

"The grate is intended to be placed as forward into the room as possible; the part in which the fire is contained is of fire-brick, the bottom being partly solid, to check the consumption of fuel. A supply of air is admitted from behind the grate, and thrown upon the top of the fire to assist in preventing smoke; the sides are splayed, so as to throw the heat, by radiation, as much as possible into the room; the opening into the chimney has no *register;* a chamber is placed behind the grate, into which air is brought from the outer atmosphere, and warmed by the large heating surface of the back of the grate, increased by flanges, and after being heated to a temperature of from 56° to 70° Fahrenheit, the air passes into the room by a shaft cut out of the wall, which terminates in a louvered opening above the reach of the men. The chamber is made as large as possible."

Fig. 5 shows the arrangement of the remodelled grate in section; and Fig. 6 shows the entire arrangement for ventilating and warming a barrack-room in the Wellington Barracks.

Such is a brief outline of the plan of ventilation suggested by the Commission, and adopted for barrack-rooms. It is simple but most promising, and is capable of wide adaptation. The Commission, it must be added, recommend that all passages, staircases, and corridors should be ventilated by shafts and perforated panes, independently of rooms. It is not to be forgotten, moreover, that "no ventilating arrangement, however perfect, can be considered as self-acting under every variety of condition," hence an intelligent, and in barracks and hospitals responsible, supervision is at all times necessary.

FIG. 5.—Section of remodelled fire-grate.

We would willingly have devoted more space to the many interesting and important suggestions and recommendations contained in the Report of the Barrack and Hospital Commission, but the foregoing illustration of their character must suffice. It is gratifying to find that, thanks to the urgent representations of the Commission, the establishment of a school at Aldershott, for the practical instruction of regimental and hospital cooks, has been sanctioned by the Minister-at-War. No reform was more urgently needed in the Army. It is gratifying also to know that, while the physical welfare of the soldier is being thus attended to, the improvement of his *morale* is not altogether overlooked. The most important step to this latter end, although approved of and supported by the War Office, is, however, a private experiment. We refer to the formation of the Soldiers' Institute at Chatham. This is intended to be the "Soldiers' Clubhouse,

**Fig. 6.—Arrangement for ventilating and warming a barrack-room in the Wellington Barracks. A A, fresh-air inlets; B (*lower*), inlet for air to be warmed in the space behind the fire grate, which air, after being warmed, passes up the flue in the wall and is admitted into the room through the louvered opening near the ceiling. The position of the chimney-flue is shown by the dotted outline; B (*upper*), the outlet foul-air shaft.**

where he will meet his comrades, smoke his pipe, drink his cup of coffee, and take his ease generally, just as his officers do at the United Service or the Army and Navy."* In the Institute, in fact, he will have all the advantages of an excellent library, reading and coffee rooms, bowling and skittle alleys and five-courts, and ultimately, it is hoped, a gymnasium, without needless restraint. The rooms are brilliantly decorated, and the building is one of the handsomest in Chatham. A large share of the government of the Institute will be conducted by non-commissioned officers and privates; and it is reasonably hoped that the care which has been devoted to the arrangements for the comfort, and in providing for the most favoured amusements of the troops, will, in some degree, wean them from the low dancing-saloons and vile dram-shops which abound in the town.

---

* *Times*, Nov. 22, 1861.

## Figures and Tables

**Fig. 1. f1:**
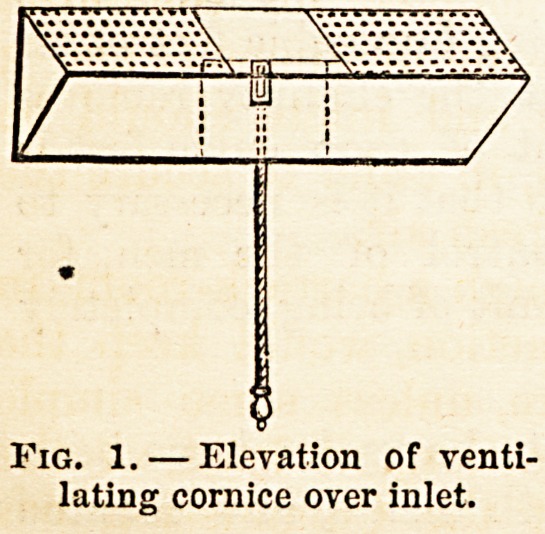


**Fig. 2. f2:**
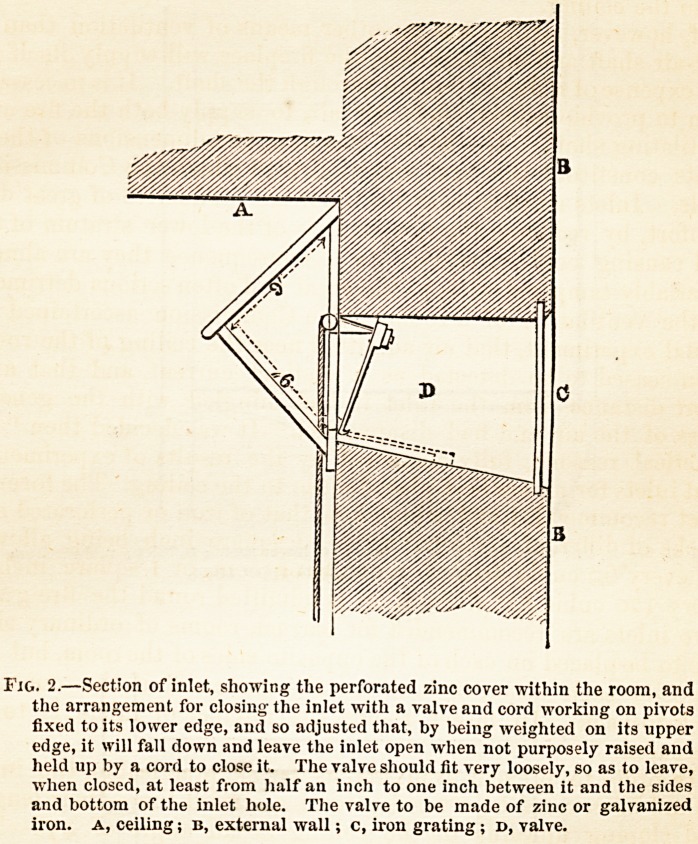


**Fig. 3. f3:**
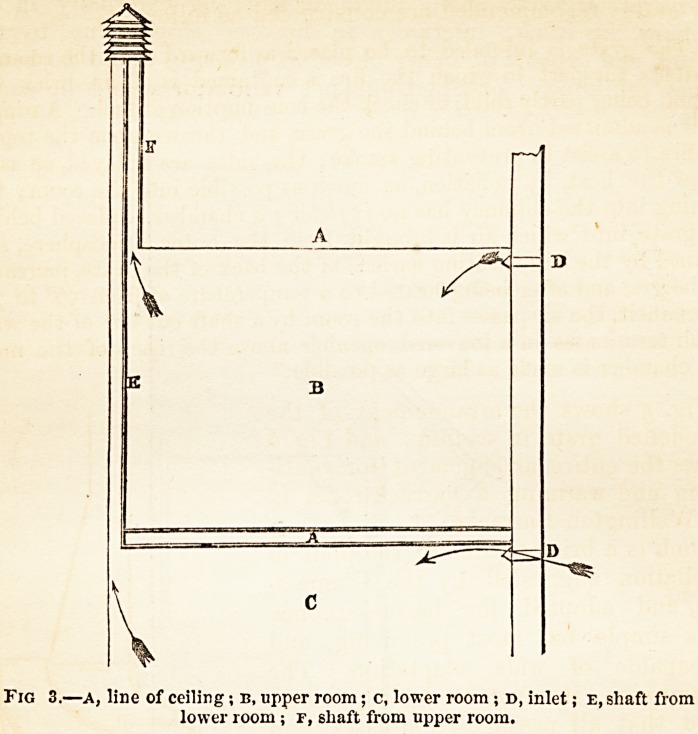


**Fig. 4. f4:**
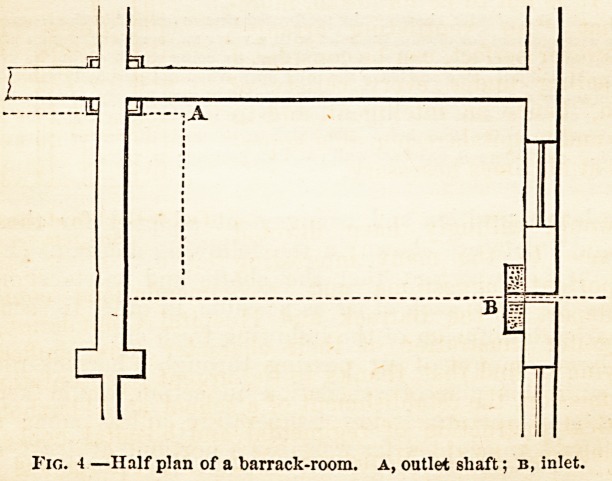


**Fig. 5. f5:**
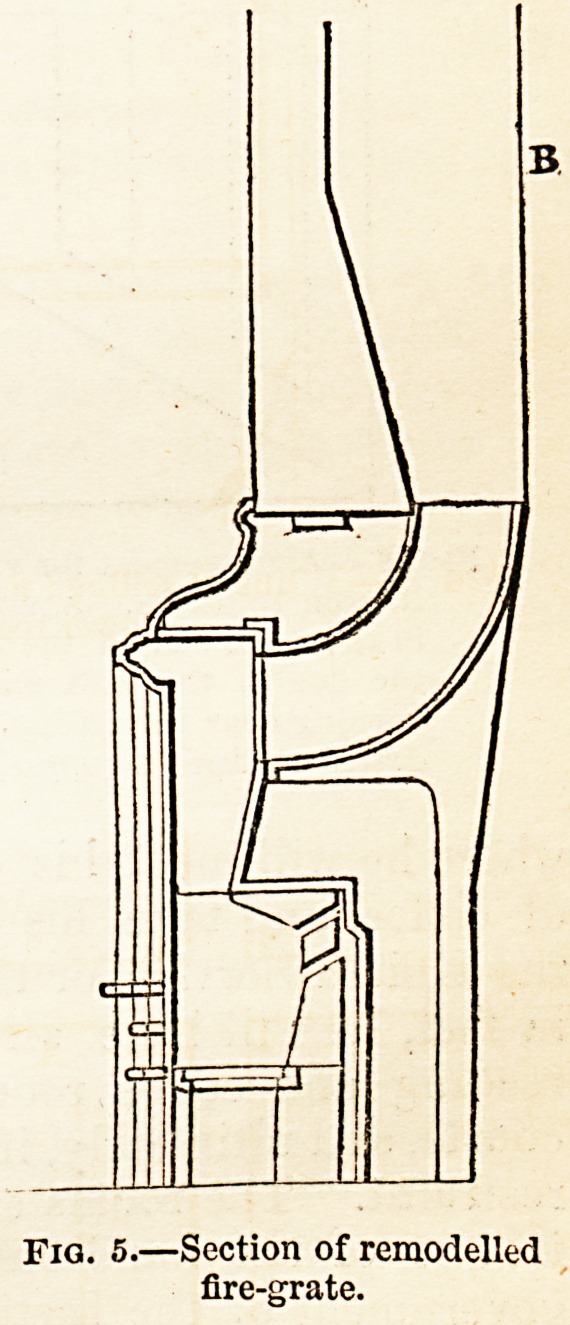


**Fig. 6. f6:**